# Using Wearable Cameras to Assess Foods and Beverages Omitted in 24 Hour Dietary Recalls and a Text Entry Food Record App

**DOI:** 10.3390/nu13061806

**Published:** 2021-05-26

**Authors:** Virginia Chan, Alyse Davies, Lyndal Wellard-Cole, Silvia Lu, Hoi Ng, Lok Tsoi, Anjali Tiscia, Louise Signal, Anna Rangan, Luke Gemming, Margaret Allman-Farinelli

**Affiliations:** 1 Charles Perkins Centre, Nutrition and Dietetics Group, School of Life and Environmental Science, The University of Sydney, Camperdown, NSW 2006, Australia; alyse.davies@sydney.edu.au (A.D.); lyndalw@nswcc.org.au (L.W.-C.); silu7263@alumni.sydney.edu.au (S.L.); hong7393@alumni.sydney.edu.au (H.N.); ltso3453@alumni.sydney.edu.au (L.T.); atis3011@alumni.sydney.edu.au (A.T.); anna.rangan@sydney.edu.au (A.R.); luke.gemming@sydney.edu.au (L.G.); margaret.allman-farinelli@sydney.edu.au (M.A.-F.); 2 Cancer Prevention and Advocacy Division, Cancer Council NSW, Woolloomooloo, NSW 2011, Australia; 3 Health Promotion & Policy Research Unit, Department of Public Health, University of Otago, 6242 Wellington, New Zealand; louise.signal@otago.ac.nz

**Keywords:** dietary assessment, nutrition, technologies, wearable cameras, young adults

## Abstract

Technology-enhanced methods of dietary assessment may still face common limitations of self-report. This study aimed to assess foods and beverages omitted when both a 24 h recall and a smartphone app were used to assess dietary intake compared with camera images. For three consecutive days, young adults (18–30 years) wore an Autographer camera that took point-of-view images every 30 seconds. Over the same period, participants reported their diet in the app and completed daily 24 h recalls. Camera images were reviewed for food and beverages, then matched to the items reported in the 24 h recall and app. ANOVA (with post hoc analysis using Tukey Honest Significant Difference) and paired *t*-test were conducted. Discretionary snacks were frequently omitted by both methods (*p* < 0.001). Water was omitted more frequently in the app than in the camera images (*p* < 0.001) and 24 h recall (*p* < 0.001). Dairy and alternatives (*p* = 0.001), sugar-based products (*p* = 0.007), savoury sauces and condiments (*p* < 0.001), fats and oils (*p* < 0.001) and alcohol (*p* = 0.002) were more frequently omitted in the app than in the 24 h recall. The use of traditional self-report methods of assessing diet remains problematic even with the addition of technology and finding new objective methods that are not intrusive and are of low burden to participants remains a challenge.

## 1. Introduction

The increasing prevalence of obesity and other diet-related diseases globally continue to make the monitoring of dietary intake an essential component to understand food and nutrient intakes of populations. Traditionally, the four dietary assessment methods of diet history, 24 h dietary recall, weighed or estimated food records and food frequency questionnaires, have been burdensome for the participant completing the intake measurements and/or for the dietitian/nutritionist analysing the food data [1].

Over the past decade, we have seen the application of technology make the process of dietary assessment less onerous [1,2,3]. A number of automated web-based and/or application (app)-based methods for the assessment of dietary intake using the 24 h recall method have been developed and validated, including Intake-24 [4,5], MyFood 24 [6] the Automated Self-Administered 24 h recall [7], Foodbook24 [8] and a web-based recall for French-Canadians [9]. To enhance self-reporting, participants are guided through structured systems to report food and beverage intake using the multiple-pass approach. The in-built design ensures a consistent method to obtain details of the food or beverage item and serve sizes and prompts for any missing items. A plethora of prospective food record apps, both commercial and researcher-designed, have also been developed using either a digital text entry food record or an image-based food record approach to record food and beverage intake [10]. The public has indicated they are ready and willing to share their data with health professionals and researchers [11]. Both the automated recalls and the food record apps have been validated using another traditional method of dietary assessment or with biomarkers such as doubly labelled water for energy, urinary nitrogen for protein, urinary sodium and other biomarkers.

However, despite the employment of technology, these methods remain self-report measures rather than objective and, as such, are still subject to participant-generated measurement bias [12]. Low energy reporting has become common in national nutrition surveys and in interventional and observational studies of nutrition [13,14,15,16]. While employing biomarkers is a more objective method to validate nutrient intakes, this technique provides a summative measure. Biomarkers cannot detect if individual meals, snacks, food or beverage groups are omitted during recording [17]. Increasingly, with a shift to studying foods consumed and dietary patterns, valid information on food intakes, not just on nutrient intakes, is required [18,19].

Wearable cameras that continuously take digital images provide an objective, first-person view of an individual’s food and beverage intake [20] and have previously been used to examine omitted foods in a small study [21]. The aim of this study was to assess the meals, snacks, foods and beverages that were omitted when both a 24 h recall method and a text entry food record app were used for assessing dietary intake in comparison with the capture of continuous images collected over a three day period.

## 2. Materials and Methods

### 2.1. Protocol

For three consecutive days, a subsample of young adults, aged 18–30 years, recruited from a large cross-sectional study [22], wore an Autographer wearable camera, recorded all foods and beverages they consumed in a researcher-designed smartphone app called EaT and Track (EaT) (The University of Sydney, Sydney, Australia) [23] and completed daily 24 h dietary recall interviews with research dietitians facilitated using the Automated Self-Administered 24 h recall Australia program (Deakin University, Melbourne, Australia) [7]. Recruitment methods are outlined in the study protocol [22]; briefly, participants had to be within the established age limits, consume at least one food item or beverage prepared outside home per week, own a Smartphone and read and write English. Participants who were pregnant, lactating or had ever had an eating disorder were excluded. The study procedures for using the EaT app and 24 h recall have been described in detail in our previous validation study [24]. The wearable camera is worn on a lanyard around the neck and captures images from a first-person perspective every 30 seconds. Participants were instructed to wear the camera for all waking hours and go about their everyday activities. The privacy lens allowed participants to halt the recording temporarily (i.e., bathroom), or the camera could be removed if individuals felt uncomfortable having their image taken [25,26]. Participants gave consent for the main study on the online initial screening and completed a basic demographic questionnaire. Those that expressed interest in the camera sub-study (24 h recall, EaT app and camera) were contacted via phone or email by the researchers and briefed about the study requirements. Demographic questions included gender (male, female or prefer not to say); age (18–24 or 25–30 years); residential postcode to determine relative socio-economic advantage and disadvantage ranking within Australia (high; top 5 deciles or low; bottom 5 deciles) [27]. Anthropometric data were collected by a questionnaire at study completion and included self-reported weight (kg) and height (cm) to calculate Body Mass Index (BMI = weight kg/height cm^2^), whose validation revealed to be acceptably accurate [28]. Deidentified camera images were stored in the university’s research data store, using only study participants’ identifier number (ID), and demographic and anthropometric information was hosted and stored in the Research Electronic Data Capture (REDCap) data management system (Vanderbilt University, Nashville, TN, USA) [29]. Ethics approval was obtained by the Institutional Human Research Ethics Committee (2016/546) on the 15 July 2016.

### 2.2. Image Coding

**Figure 1:**
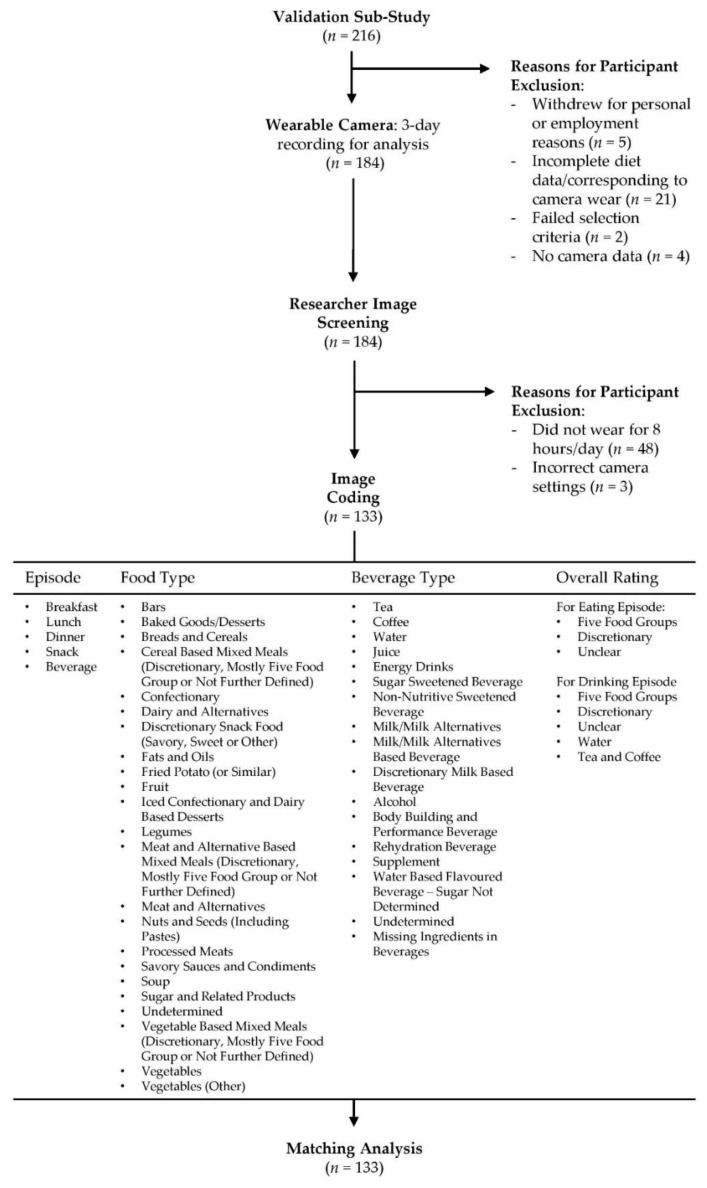
Flow diagram of the wearable camera study procedure and image coding protocol.

**Figure 2:**
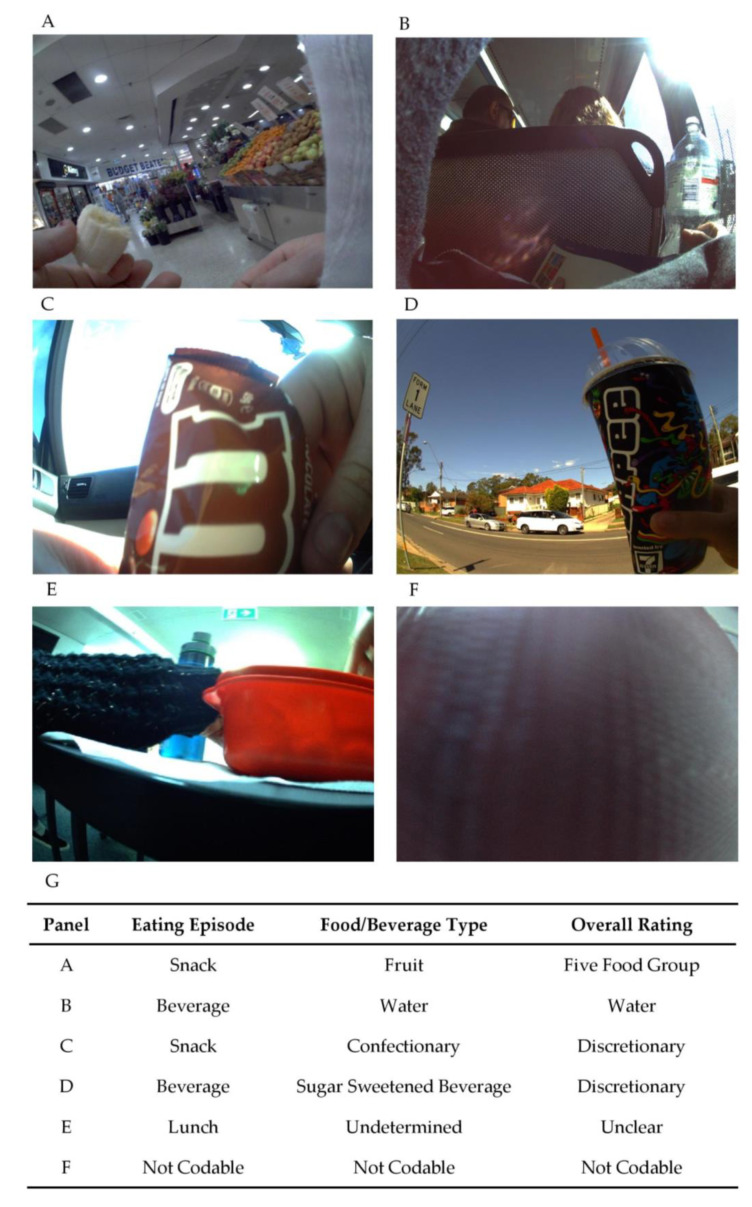
Sample image coding. Sample images depicted in panels (**A**–**F**) with corresponding codes allocated by researcher (Virginia Chan) indicated in panel (**G**).

An image-coding schedule was developed and refined using an iterative process. The coding manual is available from the corresponding author upon request. Participants (*n* = 216) recruited into the validation sub-study were screened for inclusion. Participants were excluded if they withdrew from the study for personal or employment reasons (*n* = 5), failed the selection criteria (*n* = 2), did not complete all three days of data collection (*n* = 21), did not have camera data (*n* = 4), had camera data of less than eight hours per day across the three consecutive days (*n* = 48) or had incorrect camera settings (*n* = 3). Coding commenced in March 2019 and concluded in September 2020. A total of 133 participants (487,912 images) were included in this study and were coded by an Accredited Practising Dietitian, APD, (Virginia Chan) for the consumption of foods and beverages. The dietary intake of participants was entered into Microsoft Excel spreadsheets (Microsoft Corporation, Redmond, WA, USA). All eating and drinking sequences were numbered sequentially (ascending order) for matching purposes. Figure 1 outlines the four dimensions covered in the coding schedule. The first dimension labelled all eating and drinking episodes as breakfast (i.e., first meal of the day, usually between the time of waking and 11 a.m.), lunch (typically consumed between 12 and 3 p.m.), dinner (evening meal, usually consumed between 6 and 9 p.m.), snack (any individual food item/s not identifiable as breakfast, lunch or dinner and consumed between the three meal occasions). The second and third dimensions consisted of 31 food (Supplementary Table S1) and 17 beverage (Supplementary Table S2) categories detailed below. The final dimension was the overall rating of the meal or drinking occasion consisting predominantly of five food groups (FFG) or discretionary. Two additional ratings of drinking occasions were included to report water and tea or coffee without the addition of discretionary components such as sugar, fat or confectionary. The Australian Guide to Health Eating (AGHE) was used to classify foods and beverages as either from the FFG that are recommended to comprise most food intake [30]; (1) grain (cereal) foods; (2) fruit; (3) vegetables and legumes/beans; (4) milk, yoghurt, cheese and/or their alternatives; and (5) meat, poultry, fish, eggs, tofu, nuts and seeds, or discretionary (i.e., cake, chocolate, confectionary, potato chips, pastries, sugar sweetened beverages, energy drinks, alcohol). Discretionary items were defined according to the AGHE as foods and beverages not necessary to provide nutrients and generally high in energy, saturated fats and/or added sugars, added salt, and alcohol and low in fibre [31]. Mixed meals consisting of more than one food type were classified based on the largest component (cereals, meat and alternatives or vegetables). For example, cereal-based mixed meals were meals where cereal was the major component, but all other non-cereal foods were also included in this category, i.e., all components of a pizza, including the base, cheese and toppings were included in the category of cereal-based mixed meals. See Figure 2 for an example of coding for the consumption of food and beverages. Images that were not codable for any reason (i.e., poor lighting conditions, blurry due to rapid movement or blocked by an object) were classified as not codable. Food and beverage items consumed by participants (as indicated by the use of cutlery or opaque cups) that could not be accurately determined by the coder (i.e., poor lighting conditions or obstructed camera angles) were classified as undetermined to ensure all observed eating and drinking occasions were recorded.

### 2.3. Reliability Testing

Prior to image analysis, training sessions regarding annotation rules and protocols were held to ensure coding was accurate and reproducible. A 90% agreement threshold was considered an acceptable inter-rater agreement [32]. Within the test dataset of 3557 images, model answers were generated by (Virginia Chan). Inter-rater reliability was tested with (Alyse Davies) for eating episodes (100%), food types (100%) and beverage types (92%).

### 2.4. Matching 24 h Recall or EaT App with Wearable Cameras

In total, 133 participants had EaT app and 24 h recall data matched to the camera images. Dietary data from all dietary assessment methods had a time and date stamp that were used to match the dietary intake between the 24 h recall or EaT app with the wearable camera images. The foods and beverages reported in the 24 h recall or EaT app were annotated as: (i) reported by both methods, (ii) not reported in the 24 h recall or EaT app or (iii) not identified by the wearable camera (i.e., the camera may have been turned off). For entries labelled as not reported in the 24 h recall or EaT app or not identified by the wearable camera, the omitted episode and associated food and beverage items were tabulated in Microsoft Excel. Two researchers checked all matching of data from the three sources (Virginia Chan and Alyse Davies).

### 2.5. Statistics

Descriptive statistics (percentages mean and standard deviation (SD)) were used to determine sample characteristics and the number of meals, snacks and beverages recorded by the camera per person and camera wear time. ANOVA (with post hoc analysis using Tukey Honest Significant Difference (HSD) was conducted to assess the difference between the two dietary assessment methods for meal and beverages and the camera. Paired *t*-test was used to assess differences in the number of omitted food and beverage components. Statistical analysis was conducted using SPSS software, v24.0 for Windows (IBM, Armonk, NY, USA). The significance level was set at 0.05.

## 3. Results

The demographics of the final analytical sample (*n* = 133) are shown in Table 1. The sample had a higher percentage of females (55%) than males, more adults aged 18–24 years (55%) than 25–30 years, persons in the underweight or healthy weight range (62%) than overweight or obese and of higher socioeconomic status (65%) than lower.

A total of 1822 eating occasions (main meal or snack) were identified using the wearable cameras (Table 2). Snacks were more likely to be omitted in both the 24 h recall (*p* < 0.001) and app (*p* < 0.001) compared with wearable camera images, particularly for snacks rated as comprising predominately discretionary items (24 h recall *p* < 0.001, app *p* < 0.001).

A total of 1324 drinking occasions were identified using the wearable camera (Table 2). Non-water beverages were more likely to be omitted in both the 24 h recall (*p* = 0.002) and the app (*p* = 0.002) when compared with the camera. Water beverages were more likely to be omitted in the app recording when compared with the camera (*p* < 0.001) and the 24 h recall (*p* < 0.001); this was not observed for the 24 h recall.

**Figure 3:**
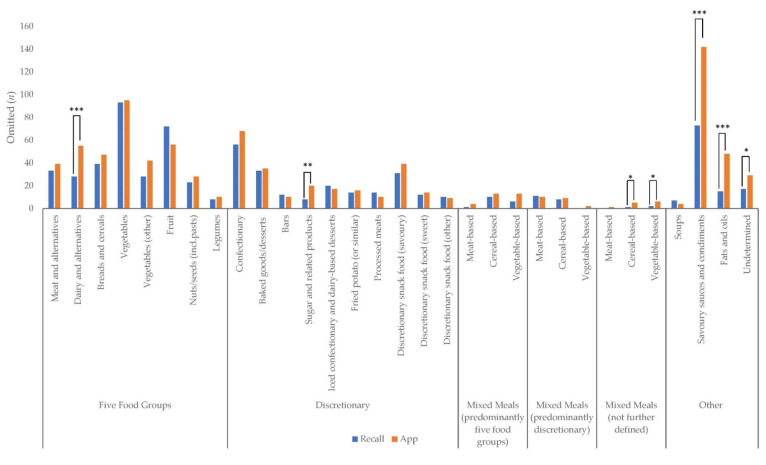
Frequency (*n*) of all food components and mixed meals omitted from dietitian-administered 24 h recall and app. Differences between 24 h recall and app that were statistically significant are indicated by bars: * *p <* 0.05, ** *p <* 0.01 ***, *p ≤* 0.001.

Figure 3 presents the frequency of all eating components and mixed meals omitted from the 24 h recall and EaT app. In total, 685 food components were omitted from the 24 h recall, and 896 from the EaT app. Significantly more dairy and alternatives (*p* = 0.001), sugar and related products (*p* = 0.007), savoury sauces and condiments (*p* < 0.001), fats and oils (*p* < 0.001) and undetermined products—i.e., items identified as consumed by the camera method though the coder was unable to assign a food category, e.g., due to poor lighting conditions or limited camera angles, (*p* = 0.039)—were omitted from the EaT app compared with the 24 h recall. Significantly more cereal-based mixed meals and vegetable-based mixed meal (not further defined) (*p* = 0.045) were omitted from the EaT app compared with the 24 h recall. The top five components omitted from the 24 h recall were vegetables (*n* = 93), savoury sauces and condiments (*n* = 73), fruit (*n* = 72), confectionary (*n* = 56) and breads and cereals (*n* = 39). The top five components omitted from the EaT app were savoury sauces and condiments (*n* = 142), vegetables (*n* = 95), confectionary (*n* = 68), fruit (*n* = 56) and dairy and alternatives (*n* = 55).

**Figure 4:**
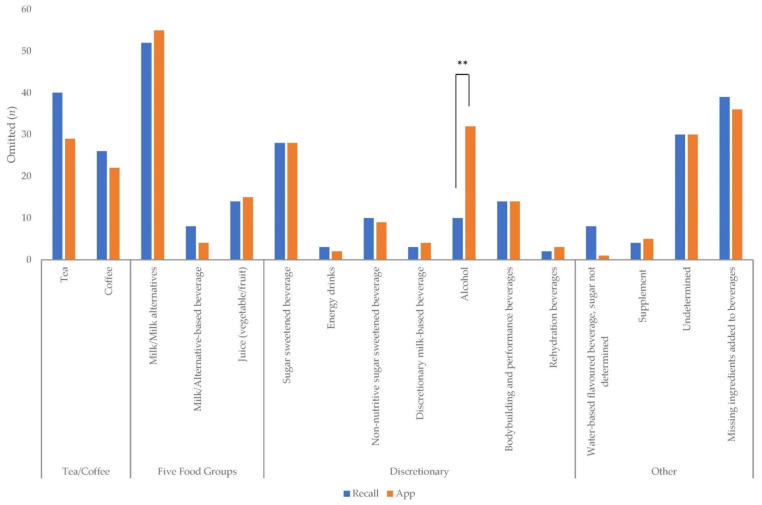
Frequency (*n*) of beverages or beverage components missing from dietitian-administered 24 h recall and app. Differences between 24 h recall and app that were statistically significant are indicated by bars: ** *p* = 0.002.

Figure 4 presents the frequency of beverages omitted from the 24 h recall and EaT app. In total, 291 beverages were missing from the 24 h recall and 289 from the EaT app. Significantly more alcohol (*p* = 0.002) was omitted from the EaT app compared with the 24 h recall. The top five beverages omitted from the 24 h recall were milk/milk alternatives (*n* = 52), tea (*n* = 40), sugar-sweetened beverages (*n* = 28), coffee (*n* = 26), juice (*n* = 14) and body-building and related beverages (*n* = 14). The top five beverages omitted from the EaT app were milk/milk alternatives (*n* = 55), alcohol (*n* = 32), tea (*n* = 29), sugar-sweetened beverages (*n* = 28) and coffee (*n* = 22). 

## 4. Discussion

The use of a wearable camera allowed for the direct observation and identification of the meals, snacks and beverages not recorded in a 24 h researcher-assisted automated dietary recall and dietary record smartphone app, both of which were designed to minimize omissions. Moreover, the study highlights the shortcomings of current self-report measures that, despite the employment of technology, are largely based on methods used in the first half of last century and prone to similar limitations [2]. The need to continue to search for better more objective methods to study the nutrition of individuals and groups is clear.

Our earlier comparison of the two dietary intake methods, which included an additional 56 participants than in this camera sub-study, revealed that participants’ app records yielded less energy intake than the 24 h recall, with a mean difference of approximately 800 kJ [24]. This is unsurprising, given that individual food items that were shown to be omitted included discretionary food and beverages that are high in saturated fat, sugars and alcohol. Sauces on meals, fats and oils, sugar-based products and alcoholic beverages were more frequently missed during app recording. All these discretionary foods are high in energy. Dairy foods, whether in tea and coffee or served with cereal, are frequently omitted food/beverage items. Similar findings have been reported by Chen et al. comparing data obtained from MyFitness Pal (MyFitnessPal Inc., San Francisco, CA, USA) to dietitian-led 24 h dietary recalls in a community setting [33]. These foods were recorded more consistently with the 24 h recall, because the multiple pass method incorporates prompts for condiments, spreads and oils added to foods during cooking or at the table. It is recommended that software developers incorporate similar features into the apps they design for the recording of food and beverages. Notably, the recall method askes about daily water consumption, while the app did not, and consequently water drinking occasions were frequently omitted. This is unlikely to be of consequence to food and nutritional analysis, unless we are trying to specifically address fluid intake. The latter may be useful in research to study how recommendations to avoid sugar-sweetened beverages might alter intakes of other beverages [34].

Another potential solution to the omission of food items in apps would be addressed by the of use image-based assessments. However, the participant must nevertheless remember to take the image to record the meal, and the addition of fats and oils as well as condiments like salt are not readily discerned. Both assessment methods missed a significant number of discretionary snacking occasions. As nutrition science moves to assess the adequacy of food intakes and dietary patterns rather than adopting a nutrient-centric approach, the omission of food groups is becoming more significant. While secondary analyses of dietary datasets can examine which food and beverages are reported less frequently or in smaller quantities between sub-groups of participants, providing indications of possible misreporting [35], these studies still cannot specifically identify what food and beverages are commonly omitted and to what degree.

The continuous camera images themselves discern eating occasions from viewing the participants’ dining experiences, from food procurement, through meal preparation and ingestion. However, image quality can be poor, and camera angles may prohibit the recognition of the food. The camera used in this research could not be recommended as providing images of a sufficient quality to recognize food and serving size in the way some of the current remote food photography methods can do [36,37,38]. One of the original intents of these cameras was to help people remember daily events [39]. Gemming et al. first used wearable camera images to examine the impact of under-reporting on energy intake using the 24 h dietary recall method, revealing omitted foods can increase self-reported energy intake by 12.5% [40]. Individuals could wear the camera to passively capture images and consult them to improve their dietary recall during the 24 h assessment. However, while continuous image capture is attractive, it is highly invasive and may raise ethical concerns if it was to be routinely employed in dietary assessment [41]. Furthermore, people may show some resistance to the uptake of such an intrusive technology in their lifestyle.

The strength of this study is the direct observation of participants’ eating habits during the period of dietary recording and recall and the meticulous coding of images for food and beverages using a well-articulated coding schedule. It must be noted this is tedious work taking 18 months and would not be recommended as routine practice until automated image recognition can be employed. It is noted that the percentage of overweight and obese participants in this study was less than that in the national statistics (i.e., 38% in the current study versus 46% for 18–24 year old individuals and 58% for 25–34 year old individuals) [42], and whether the omissions would be greater in people with obesity for social desirability reasons is questionable [43]. The limitations of this research are that the participants were able to turn off their cameras for privacy reasons and to delete private images. However, image removal was not routine. The participants’ wear time did not always encompass all the mealtimes, and consequently the current findings are likely to be an underestimation of the true food and beverage omissions. The recall method performed better than the app, but this was facilitated by a dietitian/nutritionist researcher, whereas the app recording was not. However, other research indicates that self-administration performs well compared with researcher-administered methods [44].

## 5. Conclusions

In conclusion, the use of traditional self-report methods of assessing dietary intake, even with the addition of technology, remains problematic. This research focused on young adults who mostly have high literacy with the use of technology; yet, many participants failed to record meals, foods and beverages, whether intentionally because of genuine memory loss. Researchers must continue to investigate more objective measures of dietary intake that are unobtrusive in participants lives and are of a low burden.

## Figures and Tables

**Table 1:** Sample characteristics.

Demographic Characteristic		*n* (%)
Gender	Male	60 (45)
	Female	73 (55)
Age (years)	18–24	73 (55)
	25–30	60 (45)
Body Mass Index (BMI)	<25kg/m^2, 1^	83 (62)
	≥25kg/m^2^	50 (38)
Socioeconomic status (SES) ^2^	High (top 5 deciles)	85 (65)
	Low (bottom 5 deciles)	46 (35)
**Camera Characteristics**		**Mean (SD)**
Camera wear time (h)		8.6 (1.6)
Main meals recorded by camera per person		2.5 (0.7)
Snacks recorded by camera per person		2.0 (1.3)
Beverages recorded by camera per person		3.3 (1.2)

^1^ Underweight (Body Mass Index: BMI < 18.5kg/m^2^) individuals (*n* = 3), ^2^ Socio-economic Status (SES) assessed using residential postcode to assign the index of relative socio-economic advantage and disadvantage centile employed within Australia., lowest five deciles = lower, highest five deciles = higher [27]. Two participant’s postcodes did not have an assigned decile.

**Table 2:** Number of meal and drink occasions (total and matched) over three study days as assessed by 24 h recall and app compared to meals and drinks assessed using a wearable camera.

Meal and Drink Occasions		Total Wearable Camera (*n*)	Matched (*n*)		ANOVA *p*-Value ^1^
			24 hRecall	EaT App	
Meal Episode	Main Meals and Snacks	1822	1552 ^A^	1540 ^A^	<0.001
	Main Meals	1007	969	957	0.338
	Snacks	815	583 ^A^	583 ^A^	<0.001
Main Meal Rating	Predominately FFG ^†^	698	672	671	0.727
	Predominately Discretionary	261	250	244	0.819
	Unclear	48	47	42	0.847
Snack Rating	Predominately FFG ^†^	323	247	256	0.042
	Predominately Discretionary	477	326 ^A^	318 ^A^	<0.001
	Unclear	15	10	9	0.394
Beverage Type	All Beverages	1324	1108 ^A^	1009 ^A^	<0.001
	Water	333	313	207 ^A,C^	<0.001
	All Other Beverages	991	795 ^B^	802 ^B^	<0.001
Beverage Rating ^2^	Predominately FFG ^†^	175	140	146	0.265
	Predominately Discretionary	371	296	282	0.078
	Tea/Coffee	393	328	344	0.242
	Undetermined	52	31	30	0.073

^1^ Camera, app and recall dietary method methodology assessed using ANOVA; ^2^ excluding water; ^A, B^ Statistically significant when compared to wearable cameras using Tukey HSD post hoc analysis; (A: *p*-value ≤ 0.001, B: *p*-value = 0.002); ^C^ Statistically significant when compared to 24 h dietary recalls using Tukey HSD post hoc analysis (*p*-value < 0.001); ^†^ Five Food Groups (FFG) defined by the Australian Guide to Healthy Eating [30].

## Data Availability

Data is available upon request to the authors subject to ethical approval.

## Supplementary Materials

The following are available online at https://www.mdpi.com/article/10.3390/nu13061806/s1, Table S1. Food categories and respective definitions; Table S2. Beverage categories and respective definitions.
