# Supplementary material for: Using Wearable Cameras to Assess Foods and Beverages Omitted in 24 Hour Dietary Recalls and a Text Entry Food Record App
Source: Nutrients. 2021 May 26;13(6):1806. doi: 10.3390/nu13061806 (PMC8228902; doi:10.3390/nu13061806)
Supplement: Supplementary file 1 [file nutrients-13-01806-s001.zip › nutrients-1228391-supplementary.pdf]

Supplementary Table S1. Food categories and respective definitions.

Food Type	Definition
Dairy and alternatives	Definition: Milk, yoghurt cheese and/or alternatives as defined by the Australian Guide to Healthy Eating.<br>Includes: Milk within dishes (e.g., milk on cereal), yoghurt, cheese and/or their alternatives.
Breads and Cereals	Definition: items made from grains (including: wheat, oats, rice or corn) as defined by the Australian Guide to Healthy Eating<br>Includes: All breads and cereals (with or without fruit, nuts and seeds), pasta, noodles, roti, bread rolls, flat breads, oatmeal (porridge), crumpets.<br>Excludes: breakfast cereals categorised as discretionary (> 15 g sugar per 100 g)
Fruit	Definition: All fresh fruit, diced, canned fruit, stewed and dried as defined by the Australian Guide to Healthy Eating.<br>Includes: oranges, mandarins and grapefruit, apricots, cherries, peaches, nectarines, plums, bananas, paw paw, mangoes, pineapple, melons, berries, grapes, passionfruit, apples, pears, watermelon, banana, coconut and guava<br>Excludes: fruit juice these are coded under beverages.
Vegetables	Definition: all botanically classified vegetables these can be fresh, frozen and canned vegetables.<br>Includes: Green leafy or raw salad vegetables, sweet corn, potato or starchy vegetables, tomato, celery, sprouts, zucchini, squash, capsicum, eggplant, cucumber, okra, pumpkin, green peas, green beans, potato, cassava, sweet potato, taro, carrots, beetroot, onions, shallots, garlic, bamboo shoots, swede, turnip, broccoli, brussels sprouts, bok choy, cabbages, cauliflower, kale, lettuce, silverbeet, spinach and snow peas.<br>Excludes: legumes
Vegetables (Other)	Definition: All other food items that are consumed like vegetables however botanically not a vegetable<br>Includes: avocados, olives (of any variety), mushrooms, tomatoes, Wakame (seaweed salad)
Meat and Alternatives	Definition: lean meat, poultry, fish, eggs, and meat alternatives such as tofu.<br>Includes:<br>Red meats such as beef, lamb, veal, pork, goat or kangaroo.<br>Poultry such as chicken or turkey.<br>Fish and seafood such as prawns, crab, lobster, mussels, oysters, scallops, clams.<br>Eggs<br>Tofu<br>Quorn etc.<br>Excludes: legumes, processed meats (these are separate categories)
Nuts and Seeds (incl. pastes)	Definition: Any nuts (or not botanically nuts e.g. peanuts that are consumed like nuts) or seeds. These can be non-processed, roasted, peeled, salted or made into pastes.<br>Includes:<br>Nuts such as almonds, pine nuts, walnut, macadamia, hazelnut, cashew, peanut, brazil nuts.<br>Seeds such as pumpkin seeds, sesame seeds, sunflower seeds<br>Nut/Seed Pastes such as almond/peanut butter or tahini<br>Excludes: Legume based dips
Legumes	Definition: Legumes and legume based products.<br>Includes:<br>All cooked, baked/roasted, dried or canned beans such as: red kidney beans, soybeans, lima beans, cannellini beans, chickpeas, lentils, split peas<br>Legume based dips e.g. hummus<br>Excludes:<br>Soy based products e.g. tofu that are classified under meat alternatives as sometimes it is difficult to distinguish between real and imitation meat<br>Peanuts as these are consumed more like nuts than legumes
Confectionery	Definition: Sweet discretionary products that are usually higher in sugar and/or fat.<br>Includes: Lollies, chocolate, roll-ups, nougat, fruit leather, sesame snaps, peanut brittle, chocolate coated fruit/nuts/seeds, chocolate hazelnut spreads, chocolate sauces
Bars	Definition: Product usually made from grains, whey protein, nuts and/or fruit. These can be packaged, homemade or made outside the home and purchased by the consumer.<br>Includes: Muesli bars, nut bars, protein bars/balls/biscuits/slices
Baked Goods/Desserts (homemade or similar)	Definition: These foods are usually made from flour. These may be made in an oven, microwave or on the benchtop and may have a confectionary style covering e.g. frosting or chocolate. These are usually homemade or made outside the home by a bakery, café or dessert outlet and purchased by the consumer.<br>Includes: cookies, cakes, cake-type desserts, muffins, slices, sweet pies, scones, crumbles, iced buns, pancakes or crepes with/without toppings, waffles, soufflé, croissant, fruit crumbles, pastries<br>Excludes: Bread
Discretionary snack food (Savoury)– Packaged	Definition: Packaged savoury snack foods that cannot be classified into other categories that are higher in one or more of the following: salt, sat fat, energy or added sugar.

	Examples: Potato chips, flavoured crisps and crackers, prawn crackers, popcorn, corn chips, pork crackling, pretzels, grain chips, cheese spread, rice crackers
Discretionary snack food (Sweet)–Packaged	Definition: Packaged sweet snack foods that cannot be classified into other categories that are higher in one or more of the following: salt, saturated fat, energy or added sugar<br>Examples: Biscuits with sweet fillings, Wagon Wheels, TimTams, TeeVee Snacks, YanYan, Pocky, Hello Panda etc.
Other snack food (other)	Other snack foods that do not belong to any other category.<br>Includes: Pickles, chewing gum
Iced confectionary and dairy-based desserts	Definition: milk or water-based desserts that are cold and/or frozen.<br>Includes: Ice blocks, slushies, snow cones, jelly, frozen yoghurt, patbingsoo, and icecream, gelato, sundaes, sorbet, rice pudding, fromais frais, mousse, custard
Processed meats	Definition: Meat that has been cured, salted, smoked or has had chemicals added to it.<br>Examples: Sausage, bacon, ham, salami, luncheon meats, bonito flakes, pork floss
Cereal-based mixed meal (mostly five food group)	Definition: Mixed meals where cereal or cereal products are the major ingredients AND discretionary and/or processed meats are not an ingredient which is not clearly visible.<br>Examples: Pasta, pizza, burgers, sandwiches, sushi, wraps, filled rolls, fried rice, fried noodles, ravioli, dumplings, pilaf, burrito, taco, congee (non-plain), steamed buns with fillings, nachos, risotto, gnocchi, fajitas
Cereal-based mixed meal (Discretionary)	Definition: Meals where cereal or cereal products are the major ingredients, however, are discretionary themselves i.e. are higher in fat and saturated fat, sodium and sugar or contain visible discretionary products e.g., bacon.<br>Examples: Deep fried foods, take away pizza and burgers with processed meats, sushi with tempura (fried) filling, meat pies, quiche, sausage rolls.
Cereal-based mixed meal (not further defined)	Definition: Meals where cereal or cereal products are the major ingredients and the proportion of five food group and discretionary cannot be easily discerned.
Meat or alternative based mixed meal (mostly five food group)	Definition: meals where meat and alternatives are the major constituent AND discretionary and processed meats are not an ingredient which is clearly visible.<br>Examples: Omelette, frittata, scrambled eggs, curry, stew, casserole, meatloaf, meat balls, rissoles, patties, crustless pies
Meat or alternative based mixed meal (Discretionary)	Definition: Meat or meat-based products are the major ingredients, however, are discretionary themselves i.e., are higher in fat and saturated fat, sodium and sugar or contain visible discretionary products e.g. bacon.<br>Examples: battered or crumbed fish and seafood, schnitzel, untrimmed meats (e.g., chicken wings)
Meat or alternative based mixed meal (not further defined)	Definition: Meat or meat-based products are the major ingredients and the proportion of five food group and discretionary cannot be easily discerned.
Fats/Oils	Definition: any fat-based spreads and/or oil<br>Examples: Olive oil, canola, coconut oil, vegetable oil, sesame oil, butter or margarine
Fried Potato (or similar)	Definition: any fried or oil cooked potato products including sweet potato<br>Examples: hot chips (of any thickness, cut, size e.g., French fries, wedges, thick cut), hash browns, potato gems (or tatter tots).
Vegetable-based mixed meal (mostly five food group)	Definition: A meal in which vegetables are the major ingredients AND discretionary and processed meats are not an ingredient which is clearly visible.<br>Includes: Kimchi<br>Examples: salads, vegetable-based curries, stews, or casseroles, vegetable patties, stir fries with or without meat/alternatives, dhal
Vegetable-based mixed meals (Discretionary)	Definition: A meal in which vegetables are the major ingredient, however, are discretionary themselves i.e., are higher in fat and saturated fat, sodium and sugar or contain visible discretionary products e.g., bacon<br>Examples: Tempura vegetables, deep fried vegetable patties or croquettes, Caesar salad, falafel, creamy potato bakes
Vegetable-based mixed meal (not further defined)	Definition: A meal in which vegetables are the major ingredients and the proportion of five food group and discretionary cannot be easily discerned.
Soups	Definition: All broths, blended and chunky soups based from meat, fish, poultry or vegetables, containing a variety of ingredients.
Sugar and Related Products	Definition: any form of sweetener which can be added to foods.<br>Examples: honey, sugar (caster, cane, white, raw, powdered, etc.) syrups, nutritive and non-nutritive artificial sweeteners, jam
Savoury Sauces, Condiments and Spreads	Definition: any savoury sauce, condiment, and non-fat spread<br>Examples: tomato sauce, chutney, salad dressings, mayonnaise, vinegar, (non legume) dips and yeast spreads
Undetermined	Coder is not 100% certain of the food item/product.

Supplementary Table S2. Beverage categories and respective definitions.

Beverage Type	Definition
Tea	Includes: All tea types (black, white, green, fruit tea) with or without milk/sugar/honey at any temperature (hot/iced/warm)<br>Excludes: Chain milk tea varieties e.g. pearl milk tea as these are high in sugar due to syrup used and will be considered a discretionary milk-based beverage or sugar sweetened beverages if it is a tea based beverage
Coffee	Includes: All coffee types with or without milk/sugar at any temperature (hot/iced/warm)<br>Excludes: coffee flavoured milk
Water	Includes: All water types (sparkling, mineral, still, tap, filtered) and may contain added ingredients (e.g., lemon wedge, mint, dash of apple cider vinegar) that do not significantly alter the nutritional composition<br>Excludes: Tonic Water
Juice	Includes: All fruit and vegetable juices – may be fresh, store purchased, cartooned with added or no added sugars.<br>Excludes: Juices with any additional components e.g. milk
Energy Drinks	Includes: All beverages containing caffeine, guarana or any other similar stimulant compound<br>Examples: Mother, V, Rockstar or Red Bull<br>Excludes: Coffee/Tea
Sugar Sweetened Beverages	Includes: All beverages containing added sugars and/or nutritive sweeteners.<br>Examples: Soft drinks or cordial
Non-Nutritive Sweetened Beverages	Includes: All beverages without added sugars and/or nutritive sweeteners<br>Examples: diet soft drinks, zero sugar beverages, Nexba
Water Based Flavoured Beverage – sugar not determined	Includes: Any beverage whose sugar level is not determined<br>Examples: Decanted soft drinks (coder cannot determine if it is the diet or non-diet version)
Milk/Milk Alternatives	Includes: All animal milk of all fat levels, rice milk, almond milk, macadamia milk, soy milk, drinkable yoghurts<br>Excludes: any milk-based beverages, yoghurt-based bubble tea (e.g., yoghurt and rice drink)
Milk/Milk Alternative Based Beverages	Includes: flavoured milk or milk/milk alternatives with additional five food group food items<br>Examples: Fruit smoothies, oak chocolate flavoured milk, iced coffee (without icecream or syrups), hot chocolate, milo, up and go.<br>Excludes: plain milk/milk alternatives
Discretionary Milk Based Beverages	Includes: milk/milk alternatives with additional discretionary items<br>Examples: Milk based drinks made with ice-cream, syrups and any other sweeteners e.g., chocolate milk shake with ice-cream/whipped cream or syrup, commercial milk teas with toppings (e.g., pearls)<br>Excludes: plain milk/milk alternatives or milk/milk based beverages
Alcohol	Includes: all forms of alcohol<br>Examples: Wine, whisky, beer, soju, cocktails etc.
Body Building and Performance Beverages	Includes: All sports based beverages – these are usually protein and/or amino acid based and consumed around (before/during/after) situations where the participant undertakes physical activity<br>Examples: protein shakes (made in water or milk) or branched chain amino acids (BCAAs)<br>Excludes: All electrolyte drinks because these can be consumed for purposes other than physical activity
Rehydration Beverages (Electrolytes)	Includes: all electrolyte based drinks aimed to improve the hydration status of the consumer<br>Examples: Gatorade, Powerade, Gastrolyte, HYDRAlyte, Pedialyte and Repalyte
Undetermined	Coder is not 100% certain of the food item/product
Supplements	Includes: All other oral supplements<br>Examples: Fibre supplements or meal replacement beverages or carbohydrate gels for endurance runners<br>Excludes:<br>Sports beverages and rehydration beverages<br>Meal replacement non-beverage items e.g., bars
